# Low expression of fatty acid oxidation related gene ACADM indicates poor prognosis of renal clear cell carcinoma and is related to tumor immune infiltration

**DOI:** 10.32604/or.2023.030462

**Published:** 2024-02-06

**Authors:** JIECHUAN QIU, TIANMIN YANG, YANNING SUN, KAI SUN, YINGKUN XU, QINGHUA XIA

**Affiliations:** 1 Department of Urology, Shandong Provincial Hospital Affiliated to Shandong First Medical University, Jinan, 250021, China; 2 Department of Urology, Shandong Provincial Hospital, Cheeloo College of Medicine, Shandong University, Jinan, 250021, China; 3 Department of Breast and Thyroid Surgery, The First Affiliated Hospital of Chongqing Medical University, Chongqing, 400042, China

**Keywords:** Kidney renal clear cell carcinoma, Acyl-CoA dehydrogenase medium chain, Immune infiltration, Fatty acid oxidation, Prognosis

## Abstract

This research aims to identify the key fatty acid beta-oxidation (FAO) genes that are altered in kidney renal clear cell carcinoma (KIRC) and to analyze the role of these genes in KIRC. The Gene Expression Omnibus (GEO) and FAO datasets were used to identify these key genes. Wilcoxon rank sum test was used to assess the levels of acyl-CoA dehydrogenase medium chain (ACADM) between KIRC and non-cancer samples. The logistic regression and Wilcoxon rank sum test were used to explore the association between ACADM and clinical features. The diagnostic performance of ACADM for KIRC was assessed using a diagnostic receiver operating characteristic (ROC) curve. The co-expressed genes of ACADM were identified in LinkedOmics database, and their function and pathway enrichment were analyzed. The correlation between ACADM expression level and immune infiltration was analyzed by Gene Set Variation Analysis (GSVA) method. Additionally, the proliferation, migration, and invasion abilities of KIRC cells were assessed after overexpressing ACADM. Following differential analysis and intersection, we identified six hub genes, including ACADM. We found that the expression level of ACADM was decreased in KIRC tissues and had a better diagnostic effect (AUC = 0.916). Survival analysis suggested that patients with decreased ACADM expression had a worse prognosis. According to correlation analysis, a variety of clinical features were associated with the expression level of ACADM. By analyzing the infiltration level of immune cells, we found that ACADM may be related to the enrichment of immune cells. Finally, ACADM overexpression inhibited proliferation, migration, and invasion of KIRC cells. In conclusion, our findings suggest that reduced ACADM expression in KIRC patients is indicative of poor prognosis. These results imply that ACADM may be a diagnostic and prognostic marker for individuals with KIRC, offering a reference for clinicians in diagnosis and treatment.

## Introduction

Kidney cancer is a common urinary tumor, with over 76,000 new cases of renal cell carcinoma (RCC) and over 13,700 fatalities anticipated in the United States in 2021 [1]. Kidney renal clear cell carcinoma (KIRC) is the most prevalent pathological type of renal cell carcinoma, accounting for approximately 75% of cases [2]. Because KIRC is highly insensitive to radiotherapy and chemotherapy, surgical excision is the main treatment option for early and localized KIRC. Although anti-angiogenic tyrosine kinase inhibitors and immune checkpoint inhibitors have greatly enhanced patient outcomes in metastatic patients over the last decade, most patients develop resistance [3,4]. Therefore, there is a pressing need to identify new diagnostic markers and therapeutic targets to enhance the overall prognosis for individuals with KIRC.

KIRC is a histological phenotype characterized by lipid deposition that exhibits a high degree of lipid metabolic reprogramming, and these metabolic changes influence cancer progression. Studies have shown that lipid metabolism disorders in KIRC mainly include increased synthesis of endogenous lipids, increased intake of exogenous lipids, and inhibition of fatty acid oxidation (FAO) [5]. However, current research on lipid metabolism in KIRC primarily focuses on lipid synthesis and transport, with limited attention given to lipid oxidation. Further studies have suggested that treatments for patients with kidney cancer that target FA accumulation by promoting FA oxidation may be a promising avenue for exploration [6].

The acyl-CoA dehydrogenase middle chain (ACADM) is a member of the acyl-CoA dehydrogenase family and participates in catalyzing the first step of mitochondrial fatty acid beta-oxidation. Deficiency of this gene can lead to the deficiency of medium-chain acyl-CoA dehydrogenase, which is represented by encephalopathy, fasting hypoglycemia, and liver dysfunction that can lead to infant death [7,8]. Previous researches have demonstrated the importance of ACADM in the development of glioma and hepatocellular carcinoma [9,10], but its role in KIRC remains obscure.

In our research, we utilized data from the GEO and FAO datasets to identify key genes influencing FAO. We analyzed and validated ACADM expression using TCGA data and clinical specimens and investigated its correlation with clinical characteristics. Subsequently, a survival analysis of ACADM was carried out to determine its impact on the survival prognosis of patients, and a nomogram was created to forecast the prognosis of patients with KIRC. To ascertain the specific role of ACADM in KIRC, we performed functional enrichment analysis of co-expressed genes. The connection between the level of ACADM and the infiltration state of immune cells was also analyzed. Finally, we overexpressed ACADM to deduce its function in KIRC progression.

## Materials and Methods

### Raw data download and acquisition

The Cancer Genome Atlas (TCGA) database was employed to gather the clinical data and RNA sequencing data sets for 539 KIRC tumor samples and 72 nearby normal tissues. Additionally, we acquired two KIRC RNA sequencing data sets, GSE14762 and GSE66272, from the GEO database. Thirty-seven genes related to mitochondrial fatty acid beta-oxidation were obtained from PathCards. PathCards is a comprehensive database integrating multiple human biological signaling pathways [11].

### Acquisition of clinical samples

We obtained 30 pairs of tumor samples and matched normal renal samples from KIRC patients who underwent surgical therapy at our hospital between 2019 and 2021. These 30 tissue pairs were used in this study to verify ACADM mRNA transcription levels in KIRC tissues. All patients involved in this study were informed about the purpose of the study and signed written informed consent. Our research adhered to the ethical requirements of the Shandong Provincial Hospital Affiliated to Shandong First Medical University Ethics Committee and complied with the Declaration of Helsinki.

### Differential expression

After normalization of the data from GSE14762 and GSE66272, the differential expression of mRNA in GSE14762 and GSE66272 was investigated using limma software package. The screening criterion for differentially expressed mRNAs was set to “*p* < 0.05 and| log Fold Change (FC)| > 1”, and the ggplot2 software was used to plot the volcano to show the results. The expression of screened differential genes was verified by TCGA-KIRC data set analysis. To investigate ACADM’s diagnostic efficacy in KIRC, we plotted a diagnostic ROC curve using the pROC package. To further analyze the protein level of ACADM, we obtained the protein expression differences and immunohistochemical pictures of ACADM through UALCAN and The Human Protein Atlas (HPA) databases. The UALCAN database is an interactive website with multiple features allowing users to visualize gene expression in both tumor and normal tissues [12]. It also identifies genes associated with prognosis, providing valuable tools for studying cancer in-depth. HPA database can be used to query the mRNA and protein expression of genes in different tissues, and provide users with free immunohistochemical staining results of proteins in various cancers [13].

### Venn diagram creation

Through the ggplot2 package, the up-regulated and down-regulated expression genes obtained after the differential analysis of the two data sets of GSE14762 and GSE66272 were respectively overlapped with the FAO gene set, and finally the hub gene was obtained.

### Survival analysis

GEPIA2 adds analysis of specific cancer subtypes and comparison between subtypes to the original functions of GEPIA [14]. The “Survival Map” part of GEPIA2 was employed in this research to investigate whether or not there is a correlation between the mRNA levels of six hub genes and the length of survival experienced by KIRC individuals. The Kaplan-Meier curve was utilised to assess the prognostic usefulness of ACADM in KIRC. Patients diagnosed with KIRC were divided into high-level and low-level categories according to the median expression of ACADM. The survminer and survival packages were then used to investigate the variations in prognosis that existed between the two patient groups.

### Gene enrichment analysis

LinkedOmics database is a freely accessible web tool that provides three modules for visitors to perform multi-omics analysis and visualize the results obtained [15]. In this study, 300 genes co-expressed with ACADM were obtained from the LinkedOmics online tool and analysed using the R package ClusterProfiler for Gene Ontology (GO) enrichment and Kyoto Encyclopedia of Genes and Genomes (KEGG) pathway analysis. The findings were presented using the ggplot2 package to visualise them.

### Analysis of immune cell infiltration status

Using the ssGSEA method of the GSVA package, we evaluated the link between the level of ACADM and the abundance of immune cells in KIRC individuals. Based on gene levels in cancer samples and essential genes reported in 24 immune cells, we calculated enrichment scores for each cell type. The link between the level of ACADM expression in KIRC and these immune cells was also examined using Spearman correlation analysis.

### Construct a nomogram to predict KIRC patients’ prognosis

Initially, univariate Cox analysis was employed to evaluate multiple variables, such as the expression of ACADM and clinical characteristics, in order to identify the prognostic factors influencing patient survival. Subsequently, the significant results acquired by univariate Cox analysis were analysed by multivariate Cox analysis, and independent prognostic factors affecting patients were subsequently determined. Using the final results of the analysis, a nomogram was constructed to predict the 1-, 3-, and 5-year annual survival of KIRC patients. Calibration plots were also established to show the agreement between the actual probability and the anticipated risk probability, and the 45° line represents the best prediction result.

### Cell culture with over expression plasmid transfection

Two KIRC cell lines (786-O and ACHN) were purchased from the Cell bank of the Chinese Academy of Sciences. Among them, 786-O cell lines were grown in 1640 medium (Gibco; Thermo Fisher Scientific, Inc., Waltham, MA, USA), and ACHN was grown in DMEN medium (Gibco). Both mediums contained 10% fetal bovine serum (Gibco) and 1% penicillin and streptomycin (Gibco). Both cell lines were grown in incubators containing 5% CO_2_ at 37°C. The overexpression plasmids utilized in this research were devised and constructed by keyybio (Jinan, Shandong, China). According to the experimental requirements, the tumor cells were separated into the negative control group (OE-NC group) and the overexpression plasmid group (OE-ACADM group). The cells were then transfected with plasmids according to the instructions for the plasmid transfection reagents Lipofectamine 3000 and P3000 (Invitrogen, Carlsbad, CA, USA). After ACADM overexpression efficiency was verified, plasmid-transfected renal clear cell carcinoma cells were used for subsequent experiments.

### Cell proliferation assay

The treated tumor cells were distributed at a density of 2,000 per well in 96-well plates and incubated in a cell incubator for four days. After every 24 h, the old medium was discarded, and a new serum-free medium that included 10% CCK8 (Dojindo, Kumamoto, Japan) solution was added to each well. The mixture was then placed in a cell incubator for half an hour. Finally, the absorbance (OD) of each hole at 450 nm wavelength was measured by a spectrophotometer every 24 h, and the subsequent calculation was made.

### Migration and invasion assays

The treated tumor cells were planted equally in 6-well plates for the wound healing assay, and after the cells had grown to more than 95% of the well area, the monolayer cells were scratched using a 200 μL gun tip. The tumor cells were then rinsed twice or three times with PBS to eliminate cellular debris. Each well received 1 mL of serum-free medium and was incubated for 24 h in a cell incubator. The scratches were photographed using a light microscope at 0 and 24 h. Transwell assays were performed as described in previous studies [16].

### Real-time PCR assay

First, tissue samples and cells were lysed using AG RNAex Pro Reagent (Accurate Biotechnology, Hunan, China). Next, the obtained RNA was reverse transcribed to obtain cDNA using Evo M-MLV RT Premix (Accurate Biotechnology, Hunan, China). Finally, qPCR assays were carried out with the SYBR® Green Premix Pro Taq HS qPCR Kit (Accurate Biotechnology, Hunan, China) and amplified on a LightCycler 480II (Roche). The following primers were employed in this study: ACADM-F: ACAGGGGTTCAGACTGCTATT, ACADM-R: TCCTCCGTTGGTTATCCACAT.

### Tissue microarray and immunohistochemical staining

The patients who participated in this research were KIRC patients who had surgery at Shandong Provincial Hospital. Their tumor tissues and matched normal surrounding tissues were taken for the study. Each patient was informed and signed an informed consent form. These samples were fabricated into tissue microarrays. Tissue microarrays were subjected to immunohistochemical staining following the reagent instructions. Antibodies to ACADM were purchased from Proteintech (Proteintech, Wuhan, China).

### Statistical analysis

R software (version 3.6.3) was employed to conduct all statistical analyses. Comparisons between unpaired tumors and normal tissues were made using the Wilcoxon rank sum test, whereas comparisons between paired tumors and normal tissues were made using the paired sample *t-*test. When the *p* value was lower than 0.05, statistical significance could be inferred from the data. GraphPad Prism 8.0 was used for the purpose of doing statistical analysis on the data collected from the ACADM functional experiment.

## Results

### To identify the key FAO genes in KIRC

**Figure 1:**
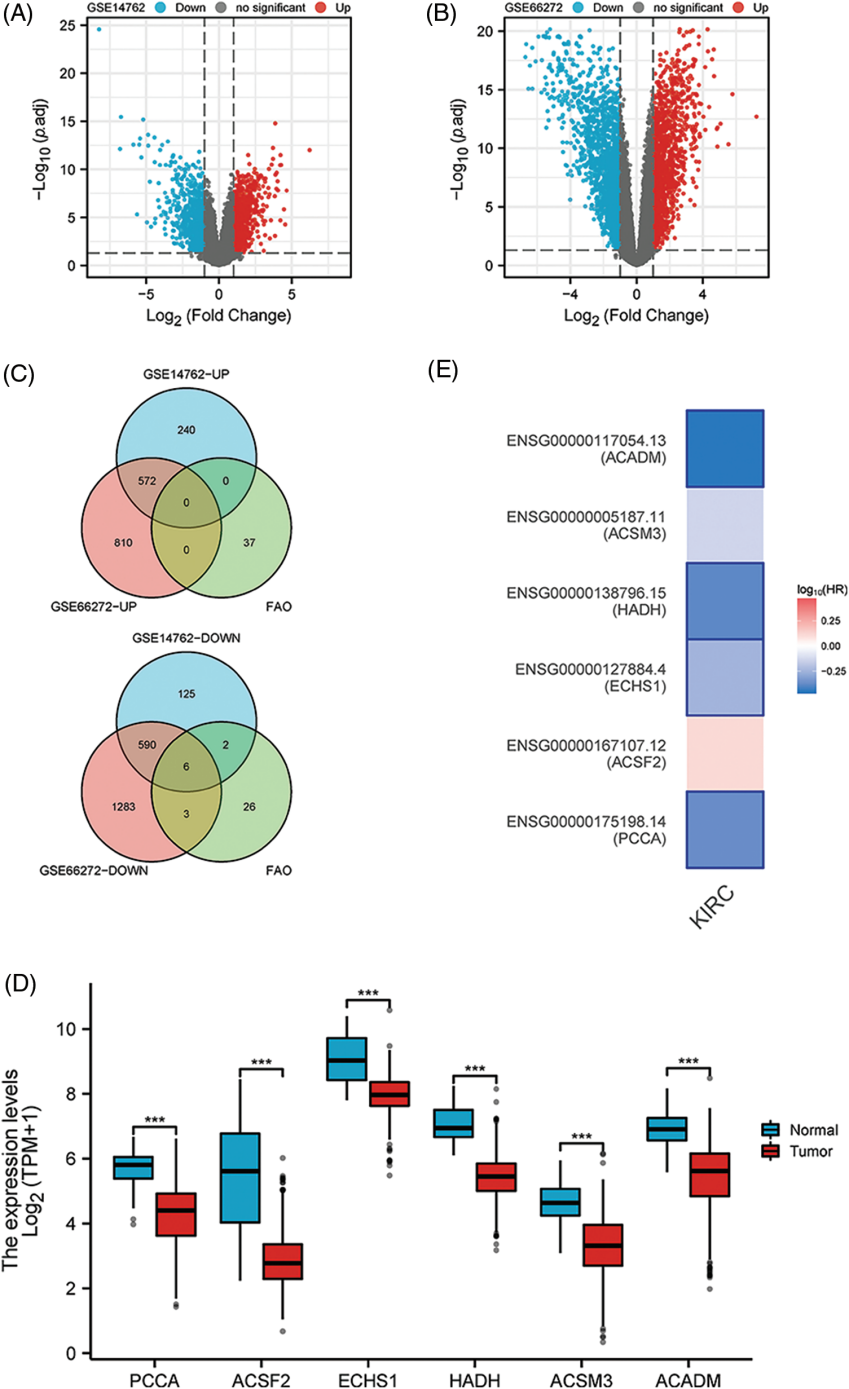
Key FAO genes were identified by the combination of GSE14762, GSE66272, and FAO datasets. (A, B) Volcano shows GSE14762 and GSE14762 differences in gene, | LogFC | > 1 and *p* < 0.05. (C) The intersection of differentially expressed genes was obtained by a Venn diagram. (D) The KIRC data set in TCGA was employed to validate the levels of 6 key genes. (E) To explore the prognostic value of 6 key genes in GEPIA2. ***p < 0.001.

After differential analysis of GSE14762, 812 genes were increased and 723 genes were decreased (Fig. 1A). Similarly, 1382 genes with elevated expression and 1882 genes with reduced expression were identified in GSE66272 (Fig. 1B). We considered the results to be statistically significant when they satisfied *p* < 0.05 and the absolute value of LogFC was greater than 1. The increased and decreased genes in the two datasets were intersected with FAO genes, respectively, and six key down-regulated genes were finally identified (Fig. 1C). Using KIRC data from the TCGA, authors confirmed the expression profiles of these six genes and found that they were all down-regulated in KIRC (Fig. 1D) (*p* < 0.001). Additionally, patients with low levels of PCCA, ECHS1, HADH, and ACADM were found to have poor prognosis in the GEPIA2 online analysis platform (Fig. 1E) (*p* < 0.01).

### Further analysis of aberrant ACADM expression in KIRC

**Figure 2:**
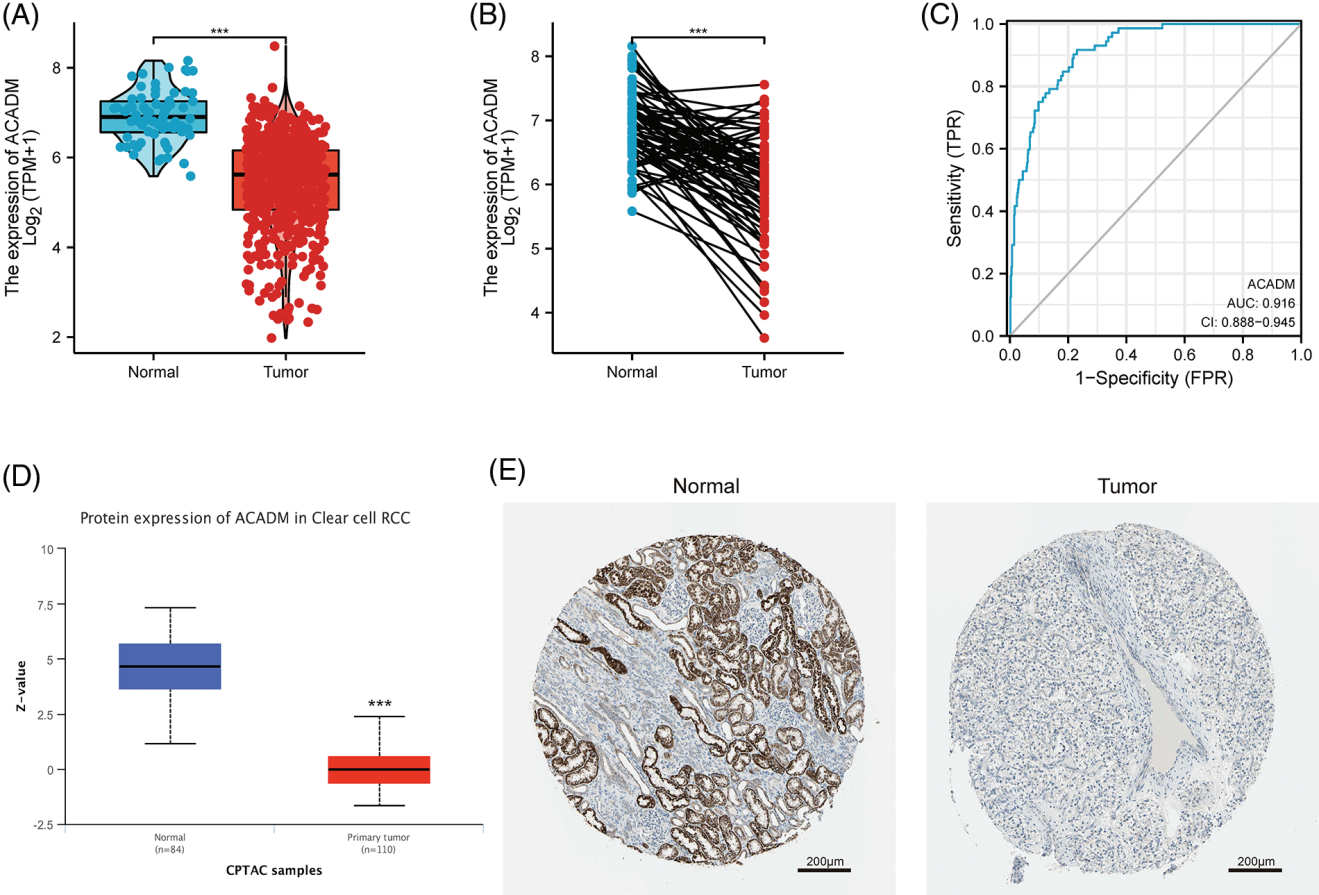
The transcription and translation levels of ACADM in KIRC have significantly decreased. (A and B) ACADM mRNA transcription levels in unpaired KIRC samples and normal kidney tissue and in paired KIRC samples and normal kidney tissue. (C) The diagnostic ROC curve demonstrated that ACADM had a good ability to distinguish KIRC tumors from normal tissues. (D and E) UALCAN and HPA databases were employed to further determine the protein translation level of ACADM. ***p < 0.001.

Currently, the functions of HADH and ECHS1 in KIRC have been studied [17,18], but the exploration of ACADM and PCCA in KIRC has not been carried out. It has been suggested that ACADM can affect fatty acid oxidation in liver cancer, so ACADM was selected for further analysis. Our analysis of KIRC paired and unpaired tissues revealed that the mRNA transcription level of ACADM was decreased in KIRC (Figs. 2A and 2B) (*p* < 0.001). We discovered that ACADM had good KIRC diagnostic effectiveness (AUC = 0.916) (Fig. 2C). Moreover, the protein expression level of ACADM was explored using UALCAN and HPA databases, and it was found that the protein translation level of ACADM was lower in KIRC (Figs. 2D and 2E).

### Correlation between ACADM expression level and various clinicopathological features of KIRC patients

**Figure 3:**
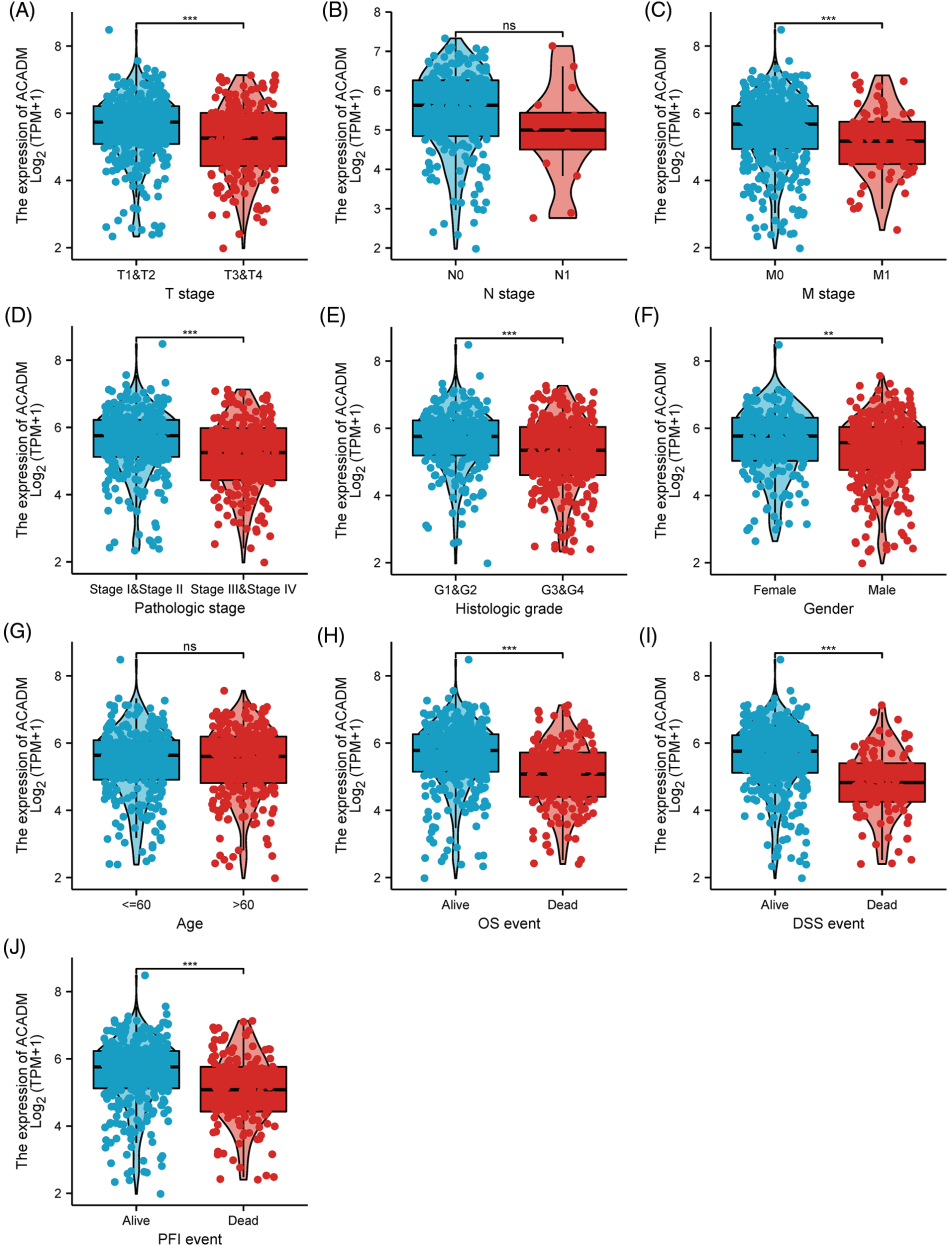
The transcription level of ACADM is correlated with multiple clinicopathological parameters. (A–C) TNM stage; (D) Pathologic stage; (E) Histologic grade; (F) Gender; (G) Age; (H–J) OS event, DSS event, and PFS event. ns, **p < 0.01; ***p < 0.001.

In KIRC patients, we evaluated the associations between ACADM transcription level and several clinicopathological features. A decrease in ACADM transcription level was discovered to be greatly linked with T stage, M stage, Pathologic stage, Histologic grade, sex, Overall Survival (OS) events, Disease-Specific Survival (DSS) events, and Progression-Free Interval (PFI) events (Fig. 3) (Suppl. Table S1). Logistic regression analysis demonstrated that ACADM transcription level was substantially linked with poor clinicopathological outcomes, including T stage (T3 and T4 *vs*. T1 and T2), M stage (M1 *vs*. M0), pathologic stage (Stage III and Stage IV *vs*. Stage I and Stage II), Histologic grade (G3 and G4 *vs*. G1 and G2), and gender (male *vs*. female) (Table 1).

**Table 1:** Logistic regression analysis was employed to analyze the link between the clinical features of KIRC patients and the mRNA level of ACADM

Characteristics	Total (N)	Odds ratio (OR)	*p* value
T stage (T3 & T4 *vs*. T1 & T2)	539	0.498 (0.346–0.712)	<0.001
N stage (N1 *vs*. N0)	257	0.325 (0.089–0.963)	0.058
M stage (M1 *vs*. M0)	506	0.397 (0.233–0.659)	<0.001
Histologic grade (G3 & G4 *vs*. G1 & G2)	531	0.447 (0.315–0.632)	<0.001
Pathologic stage (Stage III & Stage IV *vs*. Stage I & Stage II)	536	0.465 (0.325–0.663)	<0.001
Age (>60 *vs*. <=60)	539	0.964 (0.687–1.351)	0.829
Gender (male *vs*. female)	539	0.613 (0.427–0.876)	0.007

### Prognostic analysis of ACADM in KIRC

**Figure 4:**
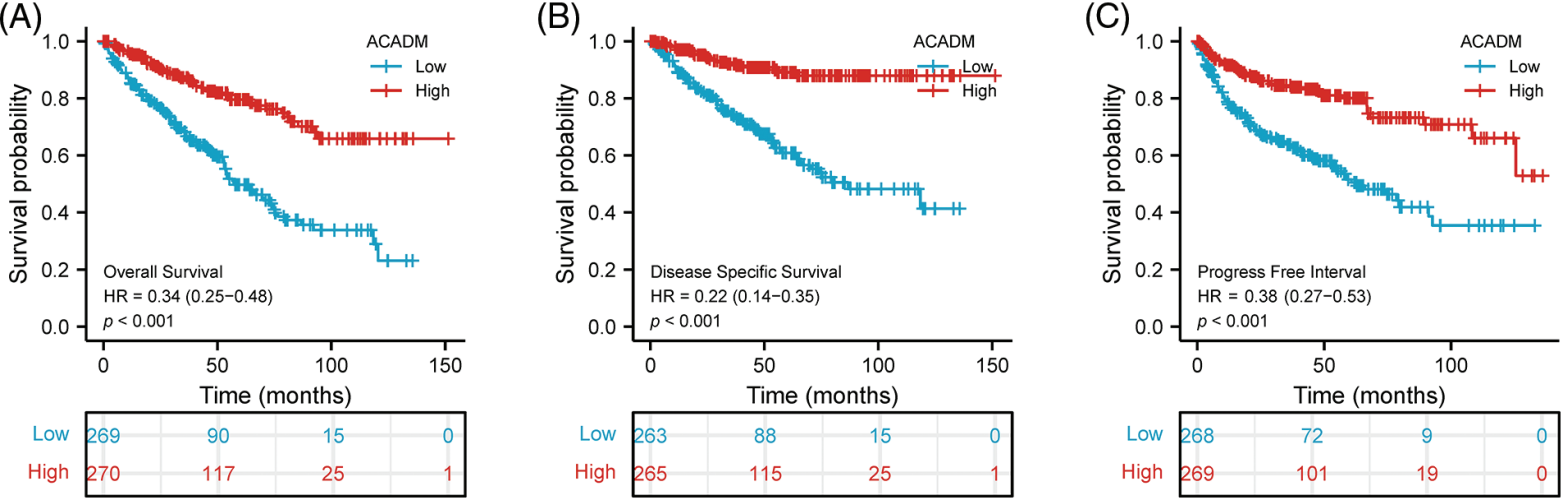
Effect of ACADM on survival outcomes in KIRC individuals. (A–C) Patients with high expression of ACADM levels showed better OS, DSS, and PFI.

**Figure 5:**
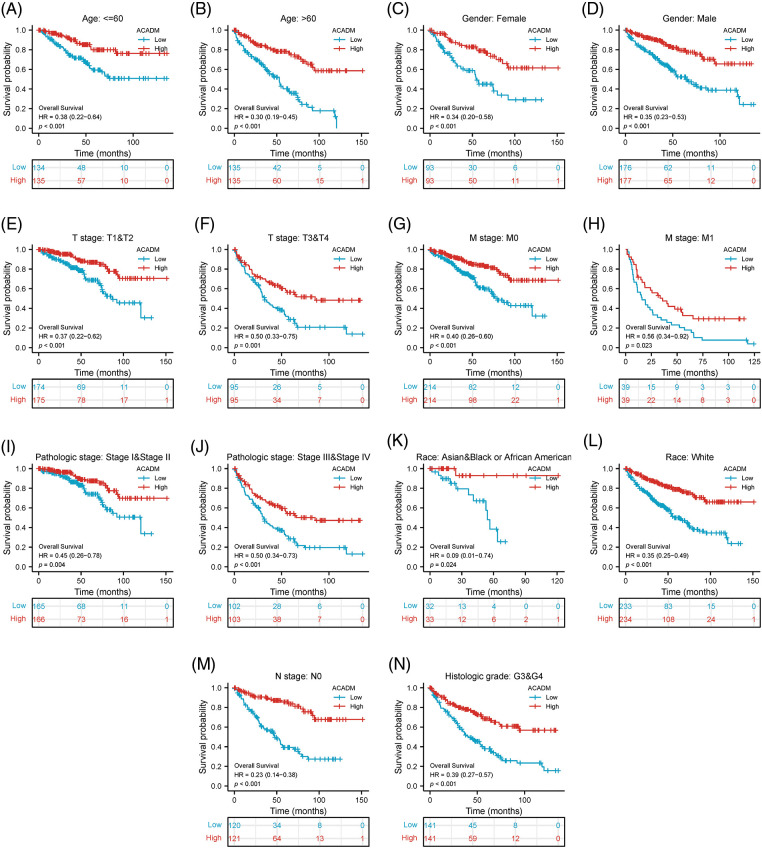
Association between the transcription level of ACADM and the survival outcomes of KIRC patients in different clinical subgroups. (A and B) Age; (C and D) Gender; (E and F) T stage; (G and H) M stage; (I and J) Pathologic stage; (K and L) Race; (M) N stage: N0; (N) Histologic grade: G3 & G4.

Using the Kaplan-Meier survival curve, the survival prognosis for patients with high and low ACADM levels was compared. In the low transcription level of ACADM patients, OS, DSS, and PFI all showed an adverse prognosis (OS: HR = 0.34, 95% CI = 0.25–0.48, *p* < 0.001; DSS: HR = 0.22, 95% CI = 0.14–0.35, *p* < 0.001; PFI: HR = 0.38, 95% CI = 0.27–0.53, *p* < 0.001) (Figs. 4A–4C). In addition, the relationship between ACADM and OS in different clinical subgroups was also analyzed. Patients with low ACADM levels have a worse prognosis in multiple clinical subgroups, including Age > 60, Age < 60, Female, Male, T1 & T2, T3 & T4, M0, M1, Stage I & Stage II, Stage III & Stage IV, race: Asian & Black or African American, race: white, N0, and Histologic grade: G3 & G4 (Figs. 5A–5N).

### Functional enrichment and pathway analysis of ACADM-related genes

**Figure 6:**
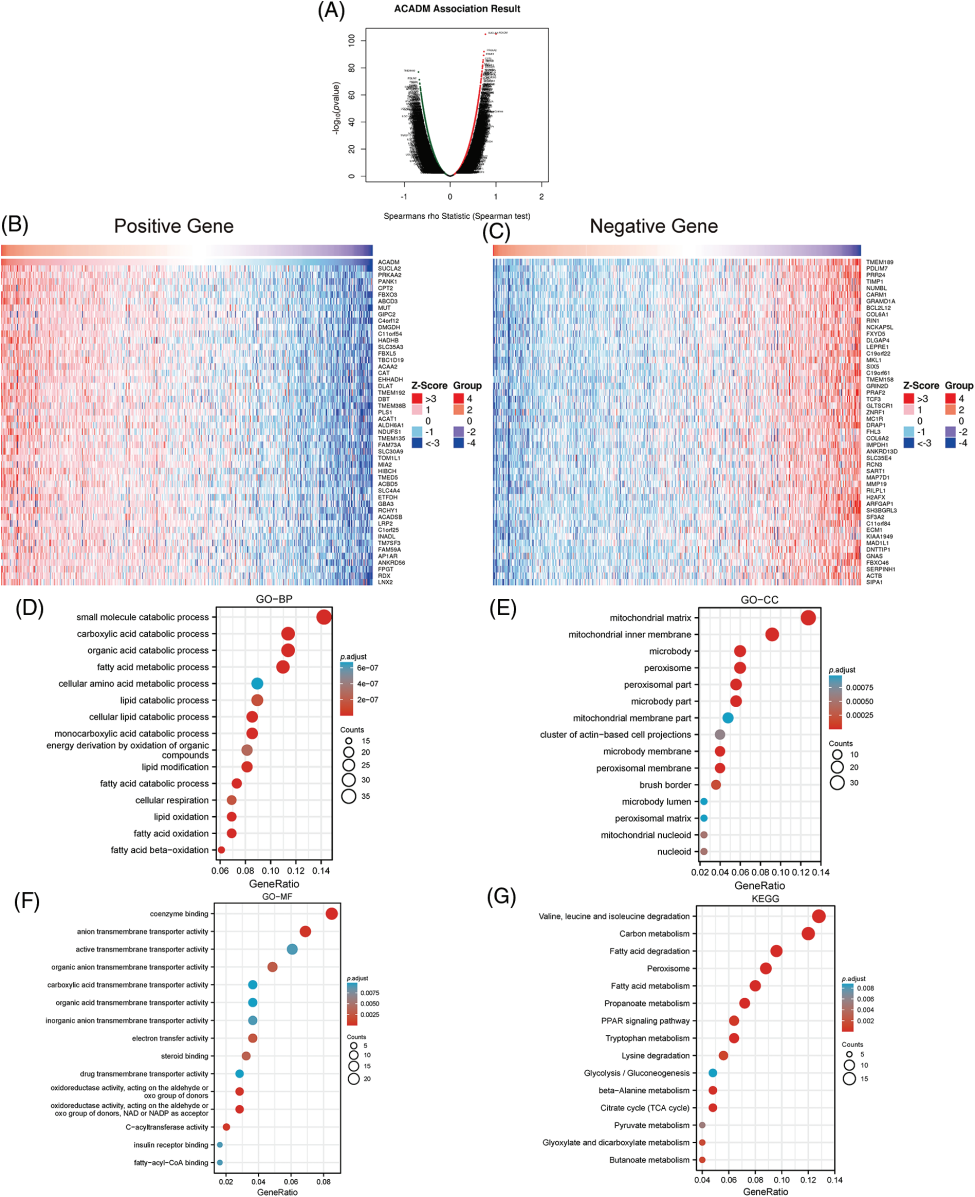
Analysis of ACADM’s functional enrichment in KIRC. (A–C) Heat maps of the top 100 coexpressed genes associated with ACADM. (D–G) GO and KEGG analysis of ACADM co-expressed genes.

As shown in Fig. 6A, the co-expressed genes associated with ACADM were obtained using the LinkedOmics online website. The 50 most positively and 50 most negatively correlated genes with ACADM were displayed by heat maps (Figs. 6B and 6C). In addition, we performed functional enrichment analysis and pathway enrichment analysis of 300 genes positively associated with ACADM. Biological processes (BP) have shown that these genes are primarily involved in small molecule catabolic process, fatty acid metabolic process, cellular lipid catabolic process, and fatty acid beta-oxidation (Fig. 6D). Cellular compositions (CC) show that the co-expressed genes were mostly involved in the formation of mitochondrial matrix, mitochondrial inner membrane, peroxisome and mitochondrial membrane part, and other cellular components (Fig. 6E). Molecular functions (MF) suggest that these genes mainly play the roles of coenzyme binding, carboxylic acid transmembrane transporter activity, and organic acid transmembrane transporter activity and fatty-acyl-CoA binding (Fig. 6F). KEGG pathway analysis suggested that pathways significantly enriched for these co-expressed genes included: Valine, leucine and isoleucine degradation, Fatty acid degradation, PPAR signaling pathway, Fatty acid metabolism, and Peroxisome (Fig. 6G).

### Construction and validation of nomogram

**Figure 7:**
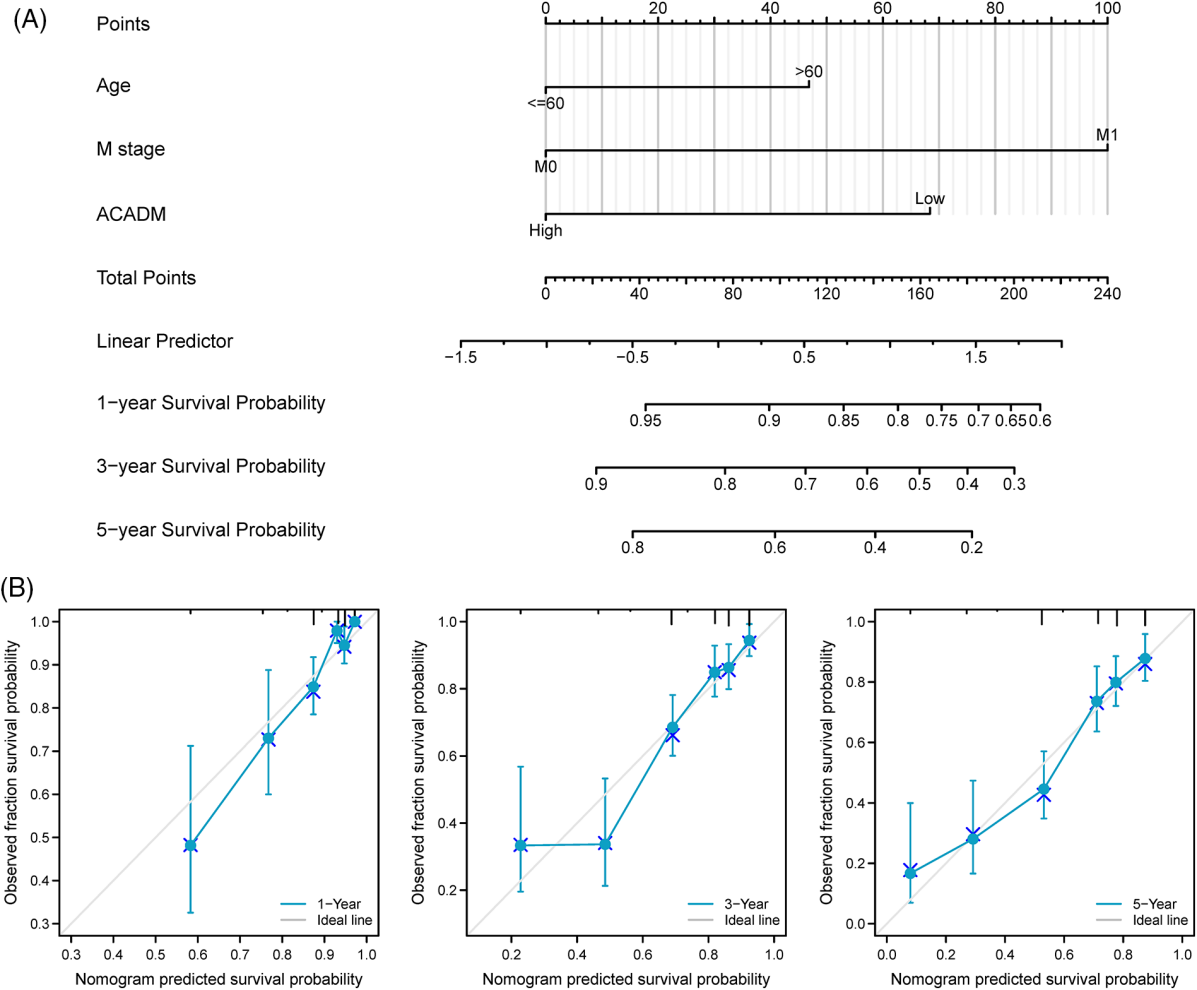
A nomogram and calibration curve for forecasting the OS of KIRC individuals were constructed. (A) Nomogram to forecast the OS of KIRC individuals in different years. (B) Nomogram calibration curves of KIRC patients in different years.

Univariate and multivariate Cox regression analysis of clinical factors and ACADM transcription level were performed to screen out separate prognostic variables, including M stage (*p* < 0.001), age (*p* < 0.05), and ACADM expression (Suppl. Table S2) (*p* < 0.001). We created a nomogram according to separate prognostic factors connected to OS to estimate the likelihood of survival at 1, 3, and 5 years for KIRC cases to forecast the prognosis of individuals with the condition (Fig. 7A). The nomogram’s computed C-index value was 0.735 (95% CI: 0.715–0.755). Furthermore, the bias alignment was found to be close to the ideal curve according to our calibration plot, indicating that these predictions were in good agreement with the actual results (Fig. 7B).

### Association between ACADM and immune infiltration

**Figure 8:**
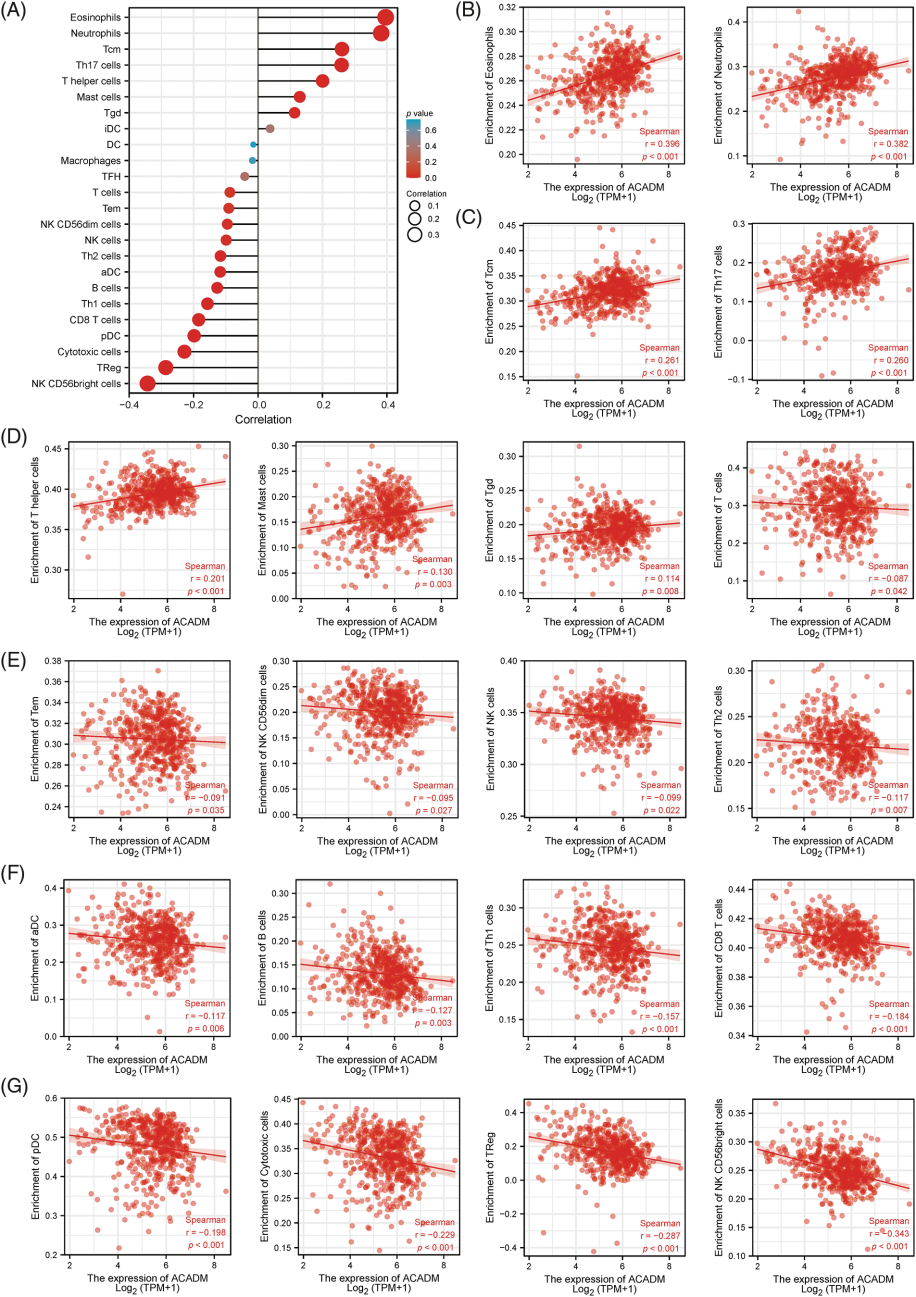
Analysis of the link between 24 immune cell infiltrating states and ACADM transcription level in KIRC. (A–G) The transcription level of ACADM is linked with the infiltration of various immune cells in KIRC.

To analyze the impact of ACADM transcription level on KIRC immune characteristics, we investigated the link between ACADM mRNA level and the enrichment abundance of 24 immune cells by Spearman coefficient. As shown in Fig. 8, ACADM levels were positively linked with Eosinophils, Neutrophils, Tcm, Mast cells, T helper cells, Th17 cells, and Tgd (*p* < 0.05). Conversely, ACADM transcription levels were negatively correlated with Cytotoxic cells, pDC, NK CD56bright cells, TReg, B cells, Th1 cells, CD8 T cells, aDC, Th2 cells, NK cells, NK CD56dim cells, Tem and T cells (*p* < 0.05).

### In vitro biological effects of upregulation of ACADM

**Figure 9:**
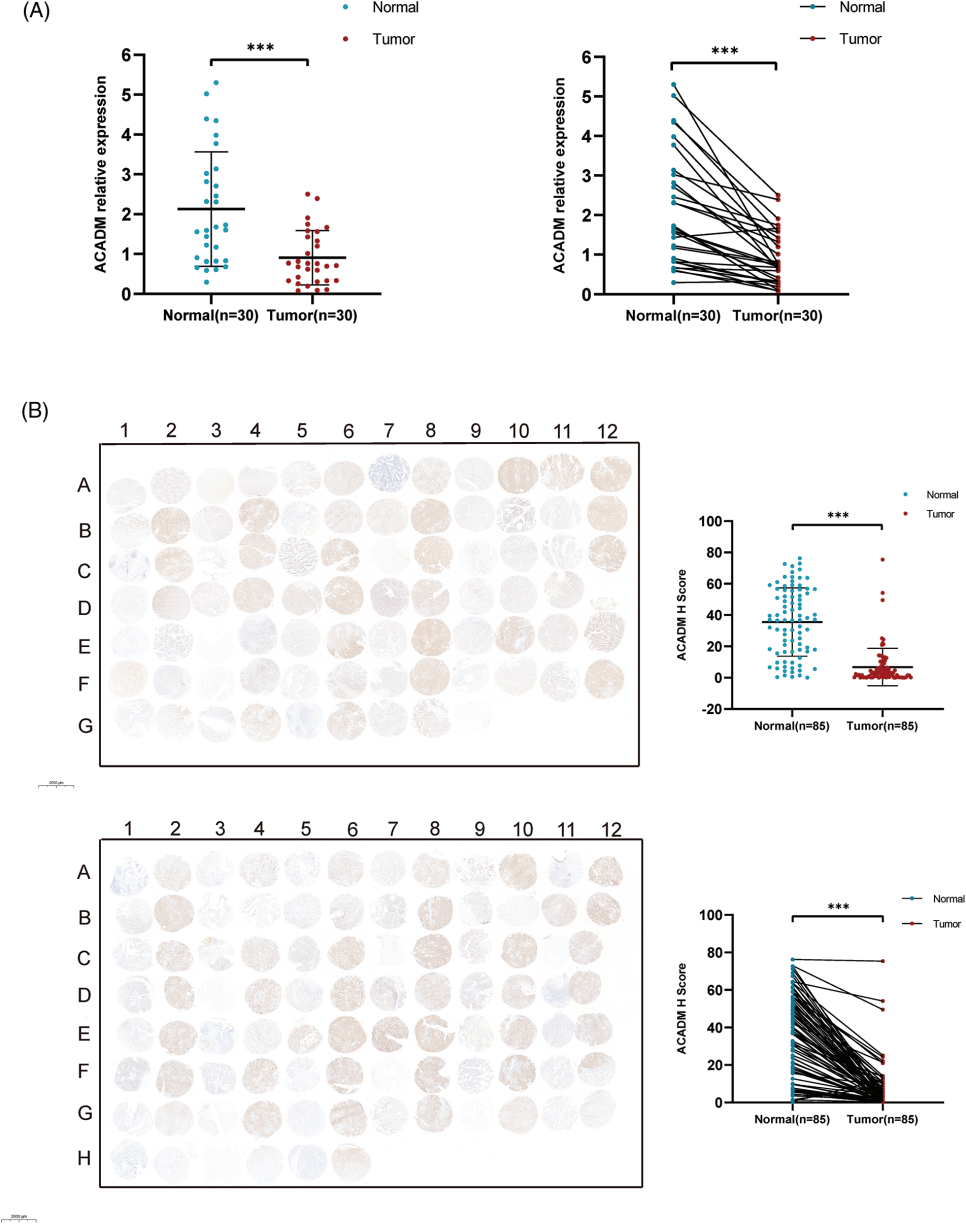
Validation of ACADM expression levels in clinical samples. (A) qRT-PCR was employed to verify the transcription level of ACADM mRNA in KIRC clinical samples (B) Immunohistochemical staining was employed to verify the translation level of ACADM protein in clinical samples. ***p < 0.001.

**Figure 10:**
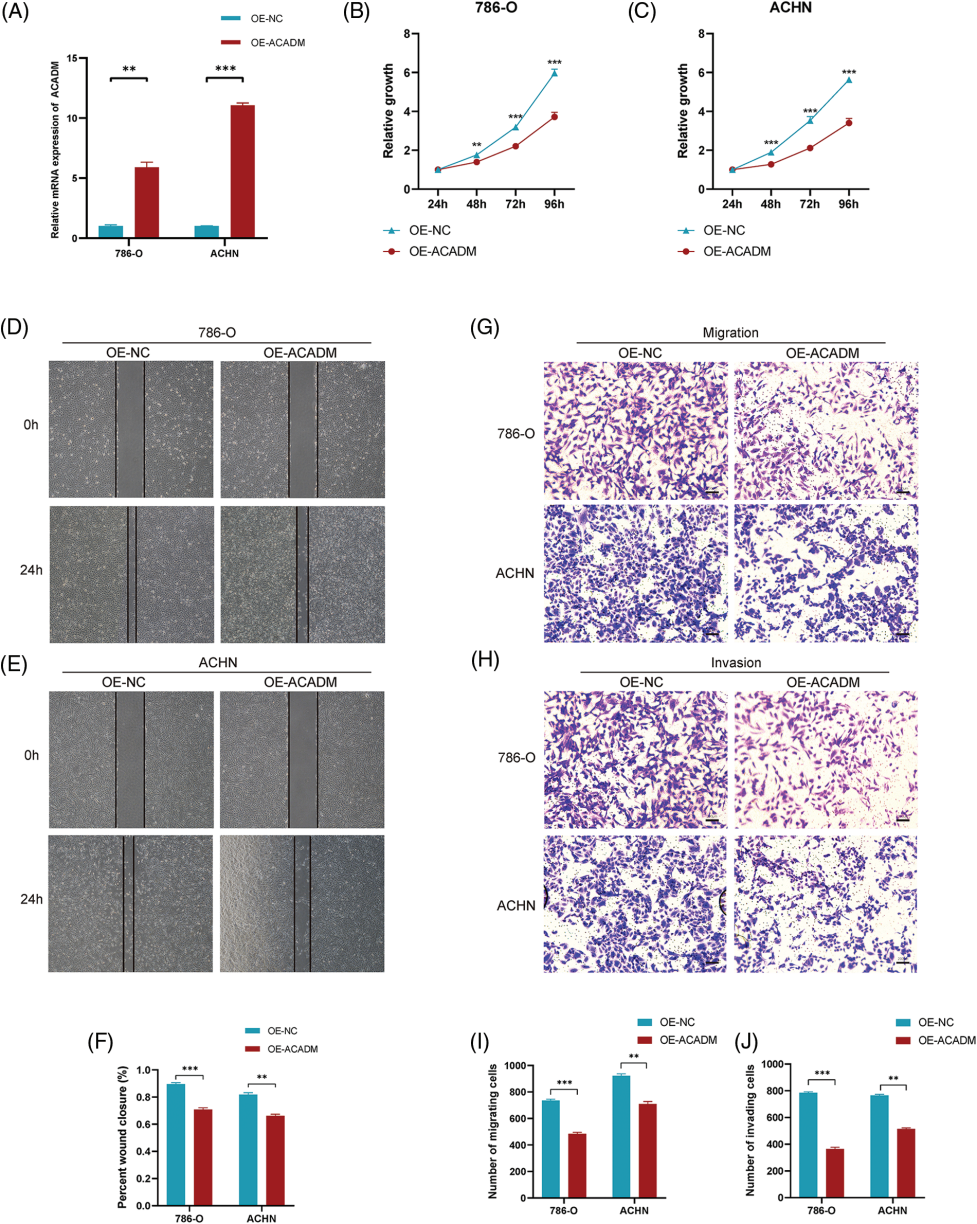
Effect of overexpression of ACADM on KIRC cells. (A) qRT-PCR was employed to detect the transcription of mRNA after ACADM overexpression. (B and C) CCK-8 assay confirmed that the upregulation of ACADM affected the proliferation of KIRC cells. (D–F) Wound healing assay confirmed that the upregulation of ACADM inhibited the migration of KIRC cells. (G–J) Transwell assay confirmed that upregulation of ACADM prevented the migration and invasion abilities of KIRC cells. **p < 0.01; ***p < 0.001.

In order to verify the expression of ACADM, we investigated the mRNA and protein levels of ACADM with qRT-PCR and IHC. The results revealed that ACADM was downregulated in KIRC samples (Fig. 9). We subsequently overexpressed ACADM in 786-O and ACHN. qRT-PCR verification found that the expression level of ACADM mRNA was increased in 786-O and ACHN after overexpression treatment (Fig. 10A). After the overexpression of ACADM, we discovered that the proliferation, invasion and migration abilities of 786-O and ACHN were greatly decreased by CCK8 assay and Transwell assay (Figs. 10B–10J).

## Discussion

As a major subtype of renal tumors, KIRC is defined by high mortality and poor prognosis. However, the treatment of advanced KIRC often gradually becomes ineffective after achieving some initial efficacy. Therefore, the discovery of novel diagnostic and prognostic biomarkers for KIRC is urgently required to serve as a guide for clinical treatment.

KIRC is currently recognized as a metabolism-related cancer that is often accompanied by metabolic reprogramming related to the metabolism of glucose, the tricarboxylic acid cycle, and the metabolism of fatty acids [19]. Among them, fatty acid metabolism has received increasing attention in KIRC. Firstly, ACC, as the enzyme that controls the rate of fatty acid synthesis, is in charge of the transformation of acetyl-CoA to malonyl-CoA [20]. According to certain research, KIRC patients have a poor prognosis when their ACC expression is high [21]. *In vitro* experiments targeting ACC have also shown that inhibition of ACC can inhibit KIRC progression. For example, it has been reported that 5-tetrokoxy2-furan acid (TOFA) (an ACC allosteric inhibitor) induces apoptosis of KIRC cells through the mTOR pathway [22]. Furthermore, patients with high expression levels of FASN protein, a central enzyme in fatty acid synthesis, had shorter OS than those with low FASN protein expression levels [23]. Secondly, enzymes related to fatty acid uptake and transport also have a crucial function in the incidence and development of KIRC. As an FA translocase, CD36 is responsible for the transport of extracellular FA into the cells. At present, studies on CD36 have shown that the expression of CD36 in KIRC is increased [24], and the elevated level of CD36 will lead to a decrease in OS and PFS in KIRC patients [25]. Mechanistic studies on the role of CD36 in KIRC showed that CD36 was upregulated by HIF-2α, and knockdown of CD36 inhibited lipid accumulation and KIRC progression [26]. FABP5, as a fatty acid-binding protein, is mainly responsible for the conveyance of FA in the cytoplasm. Previous studies have demonstrated that increased FABP5 expression in kidney cancer tissues leads to shorter OS in patients, and that when FABP5 is knocked out, the proliferative ability of tumor cells is reduced [27]. Finally, enzymes engaged in lipid catabolism in KIRC showed decreased expression. CPT1A, a crucial enzyme in FAO, is reduced in KIRC tissues as opposed to healthy kidney tissues, and its lower expression is strongly connected to shorter OS in KIRC individuals [28]. Mechanistically, CPT1A is inhibited by HIF, resulting in reduced fatty acid transport to mitochondria and promoting lipid deposition in cells [29]. When overexpressing CPT1A, intracellular lipid deposition and KIRC formation were reduced [29].

The FAO has a significant bearing on the occurrence and development of tumors. It has been found that the expression of FAO-related genes is inhibited in KIRC [30]. Enhancing FAO in KIRC has been suggested as a possible strategy for treating KIRC [5,6]. Research has shown that the expression of ECHS1 is reduced in KIRC, and its overexpression leads to reduced lipid accumulation and inhibits the progression of KIRC [17]. One study found that decreased HADH expression in KIRC patients predicted a poor prognosis [18].

In the process of fatty acid metabolism, ACADM is the enzyme that participates in the initial stage in the β-oxidation of fatty acids. ACADM is primarily responsible for the decomposition of medium-chain fatty acyl-CoA into short-chain fatty acyl-CoA and acetyl-CoA. When ACADM is deficient, it leads to hypoketotic hypoglycemia in neonates, which eventually leads to their death [31]. In HCC, the expression of ACADM is low. When ACADM expression is inhibited, intracellular lipid levels increase and promote HCC progression. Our study found that ACADM was down-regulated in KIRC, which was further verified by qRT-PCR and IHC. ACADM is also correlated with the clinical characteristics of KIRC. The level of ACADM expression decreases with increasing tumor stage and grade. At the same time, ACADM expression is also an independent prediction for OS of KIRC patients, which can be included in the nomogram to forecast the survival of patients. Functional enrichment analysis of ACADM and its associated genes showed that they were primarily enriched in fatty acid metabolic pathways. Therefore, the low expression of ACADM may lead to the attenuation of fatty acid decomposition in KIRC, thereby promoting the progression of KIRC. Studies have shown that LXR receptor agonist T0901317 can up-regulate the expression of ACADM and other enzymes, thereby inhibiting the progression of obesity in mice [32]. LXR receptor affects tumor progression by regulating glucose and lipid metabolism of KIRC [33]. Fenofibrate is a lipid-lowering drug, and several studies have found that fenofibrate has a tumor-inhibiting effect [34,35]. Recent studies have demonstrated that fenofibrate can inhibit corneal neovascularization by controlling important enzymes of lipid metabolism, including ACADM [36].

Recent studies on tumor microenvironment have shown that tumor progression may be affected by tumor immune microenvironment. The presence of immune cells among the microenvironment of cancers have a vital function during the emergence and growth of malignancies [37]. Immunotherapy targeting KIRC has also prolonged the survival time of a few individuals with advanced patients [38,39]. Treg cells perform a highly immunosuppressive function in tumors, and the increase of Treg cells indicates poor prognosis in cancer patients [40]. Research has revealed that a greater number of B cells are attracted to RCC tumor regions, and these B cells can promote RCC metastasis [41]. ACADM is believed to be one of the important enzymes participated in amino acid metabolism of colon cancer tumor-associated macrophages (TAMs), and the elevated level of ACADM is linked with a more favorable outcome for colon cancer individuals [42]. In a study of prognostic features of renal cancer according to SARS-CoV-2 related genes, a model constructed with ACADM as one of the hub genes was linked with infiltration of multiple cells of immunity [43]. According to the findings of our study, ACADM was associated with the infiltration of a number of different immune cells, including Treg cells. This indicates to some extent that ACADM may be able to alter the growth of the tumor by having an effect on the immunological milieu inside the tumor itself.

To verify the effect of ACADM on KIRC, functional experiments were performed after overexpression of ACADM *in vitro*. We found that increased expression of ACADM resulted in decreased proliferation, migration and invasion of KIRC cells. Therefore, we suggest that ACADM may have an inhibitory effect on KIRC progression.

Although this research carried out a systematic analysis of ACADM in KIRC, there are still some limitations. Firstly, authors analyzed the possible link between ACADM and cancer immune infiltration by bioinformatics methods, but unfortunately, there are not enough biological experiments to support the findings. Second, the number of clinical samples should be further increased to enhance the credibility of our research. For the influence of upregulation of ACADM on the proliferative ability of KIRC, colony formation assays were not carried out, and verification of the proliferative capacity of individual cells may be lacking. We demonstrated the inhibitory effect of ACADM on KIRC progression *in vitro*, but *in vivo* experiments were lacking to further prove it. Finally, this study only analyzed the potential biological mechanism of ACADM by bioinformatics method; however, the specific mechanism of ACADM in KIRC is still unknown.

This research demonstrates that ACADM is lowly expressed in KIRC and has a strong link with a number of clinical traits. In addition, this research also revealed the biological role of ACADM and its impact on cancer infiltration by immune cells. Our findings imply that ACADM has a significant function in predicting survival in KIRC patients. Nevertheless, further experiments are required in order to establish the precise role that ACADM plays in the development of KIRC.

## Supplementary Materials

**Supplementary Table S1:** Clinicopathological characteristics of the high and low level ACADM groups

Characteristic	Low expression of ACADM	High expression of ACADM	*p*
n	269	270	
T stage, n (%)			<0.001
T1	119 (22.1%)	159 (29.5%)	
T2	34 (6.3%)	37 (6.9%)	
T3	107 (19.9%)	72 (13.4%)	
T4	9 (1.7%)	2 (0.4%)	
N stage, n (%)			0.084
N0	119 (46.3%)	122 (47.5%)	
N1	12 (4.7%)	4 (1.6%)	
M stage, n (%)			<0.001
M0	202 (39.9%)	226 (44.7%)	
M1	54 (10.7%)	24 (4.7%)	
Histologic grade, n (%)			<0.001
G1	4 (0.8%)	10 (1.9%)	
G2	94 (17.7%)	141 (26.6%)	
G3	109 (20.5%)	98 (18.5%)	
G4	58 (10.9%)	17 (3.2%)	
Pathologic stage, n (%)			<0.001
Stage I	116 (21.6%)	156 (29.1%)	
Stage II	25 (4.7%)	34 (6.3%)	
Stage III	69 (12.9%)	54 (10.1%)	
Stage IV	57 (10.6%)	25 (4.7%)	
Age, n (%)			0.897
<=60	133 (24.7%)	136 (25.2%)	
>60	136 (25.2%)	134 (24.9%)	
Gender, n (%)			0.009
Female	78 (14.5%)	108 (20%)	
Male	191 (35.4%)	162 (30.1%)	
Age, median (IQR)	61 (53, 70)	60 (51, 69)	0.423

**Supplementary Table S2:** Univariate and multivariate Cox analyses were used to determine the relationship between clinicopathological features and OS

Characteristics	Total (N)	Univariate analysis		Multivariate analysis	
		Hazard ratio (95% CI)	*p* value	Hazard ratio (95% CI)	*p* value
T stage	539				
T1&T2	349	Reference			
T3&T4	190	3.228 (2.382–4.374)	**<0.001**	1.520 (0.662–3.488)	0.323
N stage	257				
N0	241	Reference			
N1	16	3.453 (1.832–6.508)	**<0.001**	1.308 (0.652–2.628)	0.450
M stage	506				
M0	428	Reference			
M1	78	4.389 (3.212–5.999)	**<0.001**	2.467 (1.460–4.167)	**<0.001**
Pathologic stage	536				
Stage I & Stage II	331	Reference			
Stage III & Stage IV	205	3.946 (2.872–5.423)	**<0.001**	1.245 (0.496–3.127)	0.641
Histologic grade	531				
G1 & G2	249	Reference			
G3 & G4	282	2.702 (1.918–3.807)	**<0.001**	1.366 (0.814–2.293)	0.237
Gender	539				
Female	186	Reference			
Male	353	0.930 (0.682–1.268)	0.648		
Age	539				
<=60	269	Reference			
>60	270	1.765 (1.298–2.398)	**<0.001**	1.743 (1.138–2.669)	**0.011**
ACADM	539				
Low	269	Reference			
High	270	0.343 (0.247–0.477)	**<0.001**	0.287 (0.173–0.474)	**<0.001**

## Data Availability

The bioinformatics raw and experimental data involved in this study to support our findings may be obtained from the appropriate authors for reasonable reasons.
